# The complete mitochondrial genome of *Tachysurus virgatus* (Oshima 1926) (Siluriformes: Bagridae) and its phylogenetic status

**DOI:** 10.1080/23802359.2020.1797555

**Published:** 2020-07-28

**Authors:** Yujie He, Jie Li

**Affiliations:** Pearl River Fisheries Research Institute, Chinese Academy of Fishery Sciences, Guangzhou, China

**Keywords:** *Tachysurus virgatus*, mitochondrial genome, phylogenetic analyses

## Abstract

*Tachysurus virgatus* (Oshima 1926) is a small and benthic Bagridae species that resides in the Southern China and Viet Nam. Currently, limited published genetic information has hindered our understanding of this species. In the current study, the complete mitochondrial genome of *T. virgatus* was determined using next generation sequencing, which was 16,524 base pairs (bp) in length and identical to most genomes of bony fishes. Phylogenetic analyses supported four clades (I, II, III, and IV) occurring in the family Bagridae and *T. virgatus* formed an independent clade that was sister to the clades I and II.

*Tachysurus virgatus* (Oshima 1926), a catfish species within the family Bagridae, is a small and benthic species that mainly resides in the Southern China and Viet Nam (Chu et al. 1999; www.fishbase.org). This species seems to be likely neglected due to its low economic value. To date, thus, rare genetic analyses have been conducted for this species, which limits our understanding of this species, particularly in genetic aspects. In this study, we determine the whole mitochondrial genome of *T. virgatus* for the first time using next generation sequencing technology.

The sample of *T. virgatus* was captured during December in 2019 at Zhaoqing (23.041N, 112.467E) city, Guangdong Province, China, which locates in the middle and lower Xijiang River. The specimen (voucher number: TWW2019001) was stored in the fish collection of Pearl River Fisheries Research Institute, Chinese Academy of Fishery Sciences. A bit of muscle tissues were preserved in anhydrous ethanol for DNA extraction. Total genomic DNA was extracted from muscle tissues using a Genomic DNA Isolation Kit (QiaGene, Hilden, Germany). The Illumina MiSeq platform was used to sequence the complete mitochondrial genome (Illumina Inc, San Diego, CA). We assembled the raw sequence reads into contigs using SPAdes 3.9.0 (Bankevich et al. 2012). Lastly, the complete mitochondrial genome was produced with the contigs using SOAPdenovo (Luo et al. 2012).

The complete mitogenome sequence of *T. virgatus* (GenBank no.: MT647840) is 16,524 base pairs (bp) in length and includes 13 protein-coding genes, two rRNA genes, 22 tRNA genes, and a control region. The structural organization and gene order were identical to other typical teleosts. The complete mitogenome of *T. virgatus* and 29 additional mitogenomes including 27 Bagridae species and two outgroups belonging to the family Siluridae were aligned using MUSCLE (Edgar 2004). Valid names of each species were proofread using Fishbase (www.fishbase.org). All 13 protein-coding genes were chosen by eye to combine into a single sequence for phylogenetic analyses. Bayesian inference was used for phylogenetic analyses using the optimal nucleotide substitution model (GTR + I+G), which inferred from MRMODELTEST version 2.3 (Nylander 2004). The BI analyses were performed in MrBayes 3.1.2 (Ronquist and Huelsenbeck 2003). The analyses of 20 million generations were run and sampled every 1000 generations, with the first 25% discarded as burn-in. Furthermore, neighbor-joining tree based on Kimura’s two-parameter (Kimura 1980) was implemented in MEGA 6.0 (Tamura et al. 2013) using 1000 bootstrap replicates to assess the branch support.

Both Bayesian tree and neighbor-joining tree yielded four well supported major clades with high supported values (clades I, II, III, and IV; Figure 1). Both clades I and II consisted of species from genera *Pelteobagrus* and *Tachysurus*, implying taxonomic problems occurring in these two genera. *Tachysurus virgatus* formed an independent clade (clade III) and was sister to the clades I and II (Figure 1). From the above, we suggest that more work on morphological traits and phylogenetic inferences should be combined together to better understand the relationships among Bagridae species.

**Figure 1.**
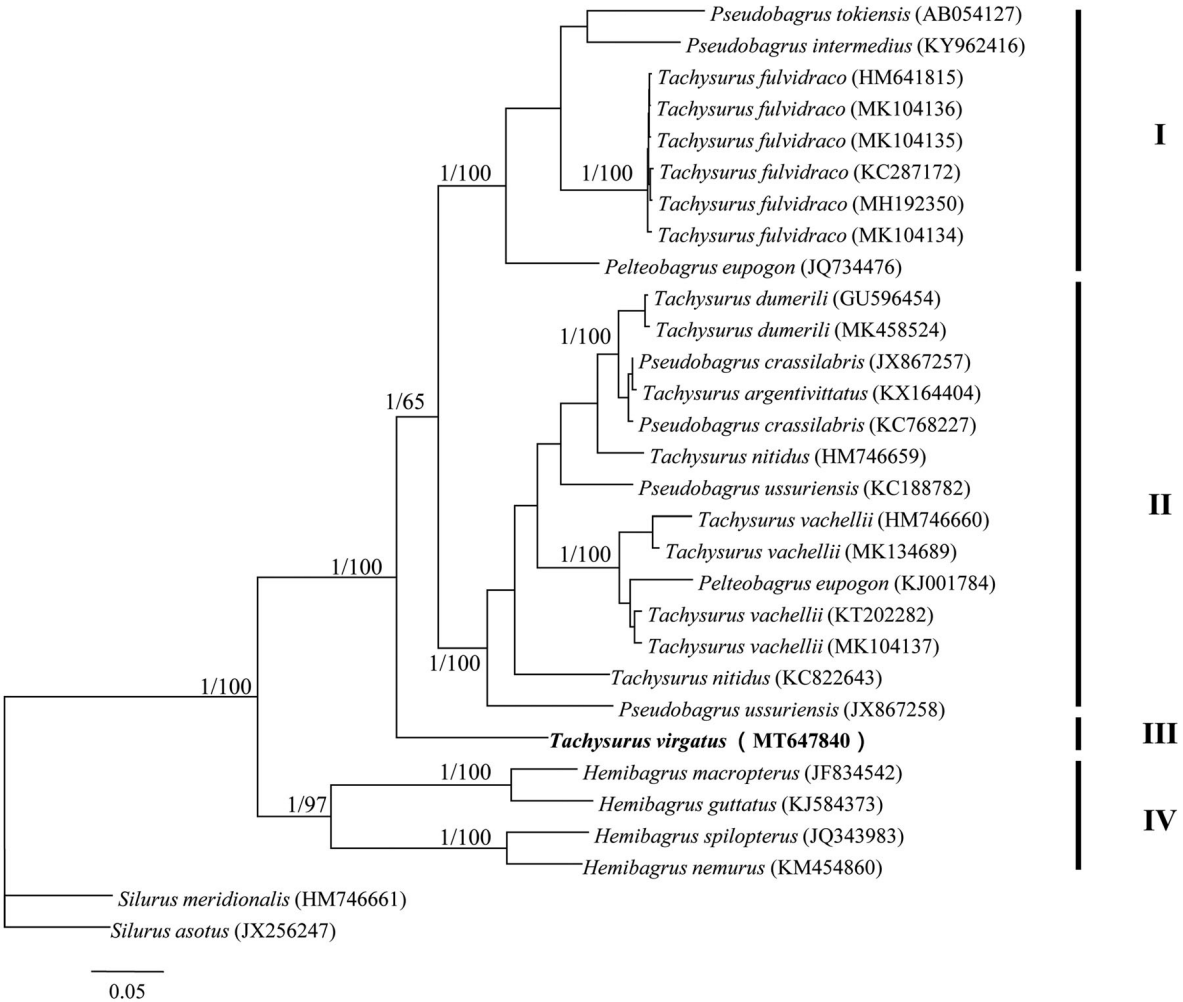
Phylogenetic tree based on Bayesian inference showing the relationships among 28 Bagridae species based on 13 protein-coding genes. Values on branches indicate Bayesian posterior probabilities and bootstrap values from a neighbor-joining analyses.

## Data Availability

The data that support the findings of this study are openly available in GenBank of NCBI at http://www.ncbi.nlm.nih.gov, reference number MT647840.

## References

[CIT0001] Bankevich A, Nurk S, Antipov D, Gurevich AA, Dvorkin M, Kulikov AS, Lesin VM, Nikolenko SI, Pham S, Prjibelski AD, et al. 2012. SPAdes: a new genome assembly algorithm and its applications to single-cell sequencing. J Comput Biol. 19(5):455–477. 22506599 10.1089/cmb.2012.0021 PMC3342519

[CIT0002] Chu X, Zheng B, Dai D. 1999. Fauna Sinica, Class Teleostei, Siluriformes. Beijing: Scientific Press.

[CIT0003] Edgar RC. 2004. MUSCLE: multiple sequence alignment with high accuracy and high throughput. Nucleic Acids Res. 32(5):1792–1797. 15034147 10.1093/nar/gkh340 PMC390337

[CIT0004] Kimura M. 1980. A simple method for estimating evolutionary rates of base substitutions through comparative studies of nucleotide sequences. J Mol Evol. 16(2):111–120. 7463489 10.1007/BF01731581

[CIT0005] Luo R, Liu B, Xie Y, Li Z, Huang W, Yuan J, He G, Chen Y, Pan Q, Liu Y, et al. 2012. SOAPdenovo2: an empirically improved memory-efficient short-read de novo assembler. Gigascience. 1(1):18. 23587118 10.1186/2047-217X-1-18 PMC3626529

[CIT0006] Nylander J. 2004. MrModeltest v2. Program distributed by the author. Evolutionary Biology Centre, Uppsala University.

[CIT0007] Ronquist F, Huelsenbeck JP. 2003. MrBayes 3: Bayesian phylogenetic inference under mixed models. Bioinformatics. 19(12):1572–1574. 12912839 10.1093/bioinformatics/btg180

[CIT0008] Tamura K, Stecher G, Peterson D, Filipski A, Kumar S. 2013. MEGA6: molecular evolutionary genetics analysis version 6.0. Mol Biol Evol. 30(12):2725–2729. 24132122 10.1093/molbev/mst197 PMC3840312

